# Natural Polymeric Composites Derived from Animals, Plants, and Microbes for Vaccine Delivery and Adjuvant Applications: A Review

**DOI:** 10.3390/gels9030227

**Published:** 2023-03-15

**Authors:** Abu Hassan Nordin, Siti Muhamad Nur Husna, Zuliahani Ahmad, Muhammad Luqman Nordin, Rushdan Ahmad Ilyas, Ahmad Khusairi Azemi, Noraznawati Ismail, Nordin Hawa Siti, Norzita Ngadi, Mohammad Saifulddin Mohd Azami, Abdin Shakirin Mohamad Norpi, Mohd Farhan Hanif Reduan, Abdinasir Yusuf Osman, Dyah Ayu Oktavianie A. Pratama, Walid Nabgan, Rumaizi Shaari

**Affiliations:** 1 Faculty of Chemical and Energy Engineering, Universiti Teknologi Malaysia, Skudai 81310, Johor, Malaysia; 2 Faculty of Applied Sciences, Universiti Teknologi MARA (UiTM), Arau 02600, Perlis, Malaysia; 3 Department of Clinical Studies, Faculty of Veterinary Medicine, Universiti Malaysia Kelantan, Pengkalan Chepa, Kota Bharu 16100, Kelantan, Malaysia; 4 Centre for Veterinary Vaccinology (VetVaCC), Faculty of Veterinary Medicine, Universiti Malaysia Kelantan, Pengkalan Chepa, Kota Bharu 16100, Kelantan, Malaysia; 5 Centre for Advanced Composite Materials (CACM), Universiti Teknologi Malaysia (UTM), Skudai 81310, Johor, Malaysia; 6 Institute of Marine Biotechnology, Universiti Malaysia Terengganu, Kuala Terengganu 21030, Terengganu, Malaysia; 7 Pharmacology Unit, School of Basic Medical Sciences, Faculty of Medicine, Universiti Sultan Zainal Abidin, Kuala Terengganu 20400, Terengganu, Malaysia; 8 Faculty Pharmacy and Health Sciences, Universiti Kuala Lumpur Royal College of Medicine Perak, Ipoh 30450, Perak, Malaysia; 9 The Royal Veterinary College, University of London, Hawkshead Lane, North Mymms, Hatfield AL9 7TA, Hertfordshire, UK; 10 National Institutes of Health (NIH), Ministry of Health, Corso Somalia Street, Shingani, Mogadishu P.O. Box 22, Somalia; 11 Faculty of Veterinary Medicine, Brawijaya University, Kota Malang 65145, Jawa Timur, Indonesia; 12 Departament d’Enginyeria Química, Universitat Rovira I Virgili, Av. Països Catalans 26, 43007 Tarragona, Spain

**Keywords:** natural polymer, vaccine delivery, adjuvant, plant, animal, microbe

## Abstract

A key element in ensuring successful immunization is the efficient delivery of vaccines. However, poor immunogenicity and adverse inflammatory immunogenic reactions make the establishment of an efficient vaccine delivery method a challenging task. The delivery of vaccines has been performed via a variety of delivery methods, including natural-polymer-based carriers that are relatively biocompatible and have low toxicity. The incorporation of adjuvants or antigens into biomaterial-based immunizations has demonstrated better immune response than formulations that just contain the antigen. This system may enable antigen-mediated immunogenicity and shelter and transport the cargo vaccine or antigen to the appropriate target organ. In this regard, this work reviews the recent applications of natural polymer composites from different sources, such as animals, plants, and microbes, in vaccine delivery systems.

## 1. Introduction

Among the most practical, economical, and long-lasting ways to protect and treat contagious illnesses is vaccination, which also slows the spread of infectious diseases. The significance of immunization by vaccination is that it trains the immune system to identify and defend against contagious diseases and provides passive community-level protection by promoting herd immunity [1,2]. Different vaccines such as DNA vaccines, synthetic peptide vaccines, nanovaccines, and recombinant protein vaccines have been created using contemporary biotechnological methods [3,4,5,6,7]. The main goal of these vaccines is to create an immunological response mediated by an antigen against the disease. Many approaches have been created to transport the vaccine to the desired place and boost the system’s effectiveness by making the vaccine more immunogenic [8]. There has been evidence of the effectiveness of a variety of delivery strategies, including polymeric, nanoparticles (NPs), and three-dimensional scaffolds [9,10,11,12]. These technologies enable the delayed release and administration of antigen molecules in a manner that does not need booster doses. Additionally, it ensures that harmful antigen molecules are effectively presented to immune cells [13]. Furthermore, an excellent vaccine delivery technology has demonstrated its capacity to function as an “adjuvant,” which interacts with the human immune system and induces an immunogenic response. Adjuvants boost the effectiveness of vaccines and decrease the number of antigens and the number of immunizations needed to produce protective immunity by enhancing the immunogenicity of immunogens that are less potent [14].

In several studies, the effectiveness and suitability of polymeric-based vaccine delivery systems for adjuvant and vaccine delivery applications were examined. The special property of polymeric-based systems is that they serve as adjuvants, may trigger an antigen-mediated immune response, and also substantially deliver the antigen or vaccine to the desired anatomical or physiological location [12,15,16,17,18,19,20,21,22]. Moreover, the benefit of polymeric-based delivery methods is that they may be made using natural polymers, which reduces the risk of tissue cytotoxicity [23]. The aforementioned points of view have been considered as we review the development of natural polymeric-based vaccine delivery systems. This review aims to examine and assess recent developments and applications pertaining to the significance of various natural polymeric-based delivery systems (from animal, plant, and microbe sources) with a focus on effective vaccine delivery and the induction of an immunogenic response that is specific to the vaccine or adjuvant. In addition, this review gives thorough insights into the immunological cascade induced by each natural polymer’s vaccine applications through its unique properties in various types of vaccines such as traditional parenteral vaccines to oral and mucosal vaccines. This review also discusses the novel applications of natural polymers for COVID-19 vaccines, which is currently the newest emerging infectious disease affecting the world for which urgent solutions are needed.

## 2. Animal-Based Polymers

### 2.1. Chitosan

Chitosan is derived from chitin, the second highly abundant polysaccharide. Chitin comprises β-(1–4)-poly-N-acetyl-D-glucosamine and can be discovered in a wide range of organisms, mostly in the exoskeletons of animals (e.g., insects, shrimp, lobsters, and crabs) [24]. The major source of chitosan was derived from removing the acetyl group (CH3-CO) mainly from chitin (poly-(β-1→4)-2-amino-2-deoxy-D-glucopyranose).

Chitosan benefits from its cationic characteristics and is a great immunomodulator in vaccine delivery, especially in the nucleic with negatively charged DNA through electrostatic interaction to produce polyplexes (2). It has been known to be used in anti-viral vaccine strategies that allow favorable interaction with the negatively charged virus. Immunotherapeutic effects of chitosan have been proved by inducing the production of cytokines (e.g., TNF-α, IL-1β, IFN-γ, and IL-10) which become a great indicator in the initiation of humoral immunity [25,26,27] and cellular immunity through stimulation of IgG antibodies [27]. Strong responses through both humoral and cellular immunity are crucial in developing a more efficient vaccine therapy.

Extensive research has been conducted on chitosan as a potential biopolymer in biomedical applications; however, chitosan presents some drawbacks such as poor solubility in physiological environments (pKa value around 6.3–6.4), and its intrinsic properties may be affected through crosslinking with other materials and potential toxicity, depending on its molecular weight and types of chitosan [24,28]. Chitosan’s anti-viral activities have been known to be impacted by these key factors: molecular weight and degree of acetylation and substitution [29]. Recently, a novel formulation of NPs of chitosan-tripolyphosphate (TPP) using the ionotropic gelation method to deliver plasmid DNA (pDNA) vaccines for cervical cancer was investigated [30]. The results showed good stability of NPs, high pDNA encapsulation efficiency rate, improved rate of cell viability during NPs cytotoxicity test in vitro, and an increase in E7 antigen transcription, the oncoprotein of high-risk human papillomavirus (HPV). This study suggested a good formulation for nucleic acid vaccine vehicles for viral infection and others.

The issue of muti-drug resistance in tuberculosis (TB) [31] adds additional urgency to developing a new vaccine formulation, which is currently restricted to the BCG vaccine only. Several studies have attempted to produce new formulations for TB vaccines by using chitosan-based NPs. A novel nanosphere of chitosan (CHT)- or trimethyl chitosan (TMC)-coated PLGA (i.e., containing HspX, EsxV, and PPE44 *Mycobacterium Tuberculosis* (*Mtb*) antigens) was designed [32]. Hspx–PPE44–EsxV (HPE), together with the adjuvant of resiquimod (RC), HPERC, was loaded in chitosan in a study regarding the TB booster vaccine to overcome waning immunity in TB [33]. Both studies showed a Th1-dominant response in groups incorporated with chitosan polymers and groups with single BCG immunization and HPERC vaccine booster groups compared to control groups. These studies present a good vaccine candidate for TB vaccination. Delivery of vaccine through a transdermal route using microneedle (MN) patch aid with the iontophoresis technique can monitor the transportation of vaccine molecules. However, MN serves a limitation such as a low load of vaccine; therefore, a polyacrylamide/chitosan iontophoresis-driven MN system was designed to deliver ovalbumin, and it resulted in enhanced immune response compared to conventional intramuscular injection in vitro [34].

**Figure 1:**
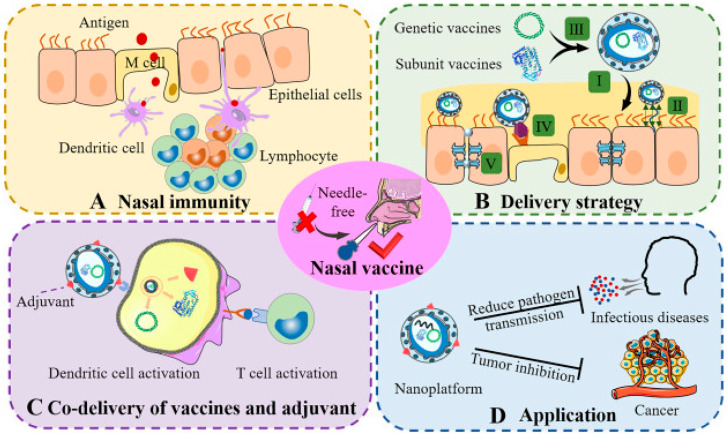
Strategies for nasal vaccine delivery. (**A**) Representation of nasal immunity. (**B**) A delivery strategy for nasal vaccines using genetically modified vaccines or subunit vaccines. (**C**) Action of co-delivery of vaccines and adjuvant. (**D**) Effects of nanoplatforms in medical applications. Adapted from Teng et al. [37].

Moreover, mucosal vaccine administration (e.g., through oral, nasal, and vaginal routes) is more favorable compared to parenteral administration due to several factors such as local immune response activation and eliciting epigenetic memory, which can induce greater immunogenicity during second exposure [35,36]. Figure 1 illustrates the strategies for nasal vaccine delivery.

Chitosan was used in various applications as a basis for mucosal vaccine delivery vehicles due to its mucoadhesive characteristic. Goblet cells in the large intestines release mucus so that chitosan is able to hydrogen bond with the mucin of the mucus by retaining OH and NH2 groups when at physiological pH (6 to 7.5), serving an excellent deal in mucosal vaccine delivery. Table 1 shows the novel studies on chitosan-based mucosal vaccine delivery vehicles.

Chitosan also can be applied as an adjuvant for vaccines to boost the immune system. Chitosan derivatives are more favorable compared to an unmodified form of chitosan. Some recent studies on chitosan adjuvants are as follows. Chitosan-modified as the carrier for adjuvant nanographene oxide (GO-CS) and P239 vaccine for hepatitis E virus (HEV) treatment [26]. Adding chitosan as an adjuvant improved graphene oxide toxicity and increased bioavailability. In comparison with the P239 vaccine alone, the GO/CS/P239 vaccine induced more production of IgG antibodies and activation of cytokines.

A combined adjuvant system was achieved by encapsulating chitosan and *Salmonella Typhi* porins in micro (MicroAS) and nanoparticulate (NanoAS) forms to carry rGRA1 and rBAG1 vaccine antigen against *T. gondii* in Toxoplasmosis disease was presented [27]. The new formulation resulted in higher cellular and humoral response in the microsystem compared to the nanosystem. Thus, this formulation is more efficient in microsystems by profoundly increasing the protection against *T. gondii.* Readers are directed to a more detailed explanation of the application of chitosan as an adjuvant in the following article [44].

In summary, chitosan has shown great efficacy in vaccine formulation, both as a carrier and adjuvant. These easily available natural polymeric materials are foreseen as excellent biomaterials in the COVID-19 vaccine delivery platform [45], exploiting various capabilities in mucosal vaccine delivery in various viral infectious diseases shown in Table 1. In addition, chitosan’s unique properties as a natural mucoadhesive material are critical to prolonging antigen retention and tissue penetration through its ability to temporarily open intercellular tight junctions that are beneficial to amplify both humoral and cellular immunity in chitosan-based vaccines where broad studies have been conducted to apply this biomaterial as vaccine delivery and adjuvants for various diseases. Overseeing the potential of chitosan application in mucosal vaccination presents a good approach for COVID-19 vaccination as its viruses enter the host through the mucosal as the main route. This will be further discussed in Section 5. A summary of chitosan-based polymers for vaccine delivery and as adjuvants is presented in Table 2.

### 2.2. Gelatin

Gelatin is a form of soluble protein that is derived from insoluble fibrous protein, collagen (i.e., main component of extracellular matrix in animal tissues such as skin cartilage and bone) [46]. Pre-treatment of collagen using acid treatment and alkaline treatment resulted in type A and type B gelatin, respectively. This biopolymer is characterized by a variety of accessible functional groups, hydrophilic biodegradable polypeptide polymers, that serve a promising window in the encapsulated high payload of antigens and have been known to be successfully used in the vast application of vaccine delivery systems [47].

Apart from that, gelatin also can be modified into coupling with many cross-linkers [47,48] and low antigenicity [49], making it a well-established vaccine delivery vehicle. The potential of gelatin can be seen through a study that showed human macrophages were successfully targeted when observed during the application of gelatin hydrogel nanofiber where this system provided great immunomodulatory effects by upregulating the pro-healing gene of M2 macrophage (CD206) and markedly reducing the pro-inflammatory gene (IL-1β and IL-8) expression [50]. Macrophage-mannose receptor CD206 was characterized as a negative-prognostic biomarker for many malignancies where this biomarker improves the overall survival (OS) of the patients [51,52,53]. This indicates a positive impact of gelatin to induce CD206 to be incorporated as a vaccine for cancers.

The malaria (i.e., one of the top three infectious diseases worldwide) vaccine is not commercially available due to the vast development stages of *Plasmodium* in host and immune responses making malaria vaccine production a major challenge [54,55]. Plus, effective vaccine delivery tools are demanded, especially in endemic areas with poor healthcare facilities. Surface protein P47 of *Plasmodium falciparum* and adjuvant CpG was fabricated into gelatin-based MN, where this system triggered toll-like receptor (TLR) 9 (TLR9) signaling and dendritic cells (DCs) at the same levels achieved by the native vaccine, thus showing the potential of the P47 MN-gelatin-based malaria vaccine that could be deployed [56]. In another study, aminated nanoparticulate MN-based gelatin resulted in a higher production of antibodies against tetanus toxoid compared to gelatin NPs [57]. For the synthesis of gelatin NPs, Khramtsov et al. [58] proposed a one-step modified desolvation method to overcome the limitations of the common two-step desolvation method associated with poor reproducibility and low yields. This method presented the reproducible synthesis of gelatin NPs, with yields of 62–82%.

The result of a study showed that gelatin NPs were convenient carriers for the delivery of antigens OVA and adjuvant polyinosinic: polycytidylic acid in various administrations, such as through mucosal delivery, where they both can modulate systemic and mucosal immune responses, including EG7 tumor growth inhibition in C57BL/6 mice [59]. Gelatin NPs have been studied to simultaneously deliver messenger RNA (mRNA) and plasmid DNA (pDNA) [60]. Transfection efficiency was observed in polynucleotides indicating a potential system using gelatin in vaccination deployment. Mannosylated gelatin NPs encapsulated inactivated porcine reproductive and respiratory syndrome virus (PRRSV)-induced activation of T cells in vitro through triggering DC maturation and activation, consequently inactivating PRRSV-infected cells via the improved T-cell signaling cascade [61].

A vaccine against botulinum toxin was fused into gelatin-based MN vaccines, resulting in good immunogenicity and protection efficacy of the AHc vaccine when stored at room temperature for 6 months, which indicates a brilliant vaccine strategy, especially in overcoming the limitation of the vaccine in terms of the cold-chain problem [62]. Taking advantage of various accessible functional groups that can be targeted by gelatin serves as a great opportunity in new vaccine development, especially for uncommercially available vaccines such as the malaria vaccine, which is still scarce and poorly accessible. Plus, MN-based gelatin has great potential to deliver high-molecular-weight vaccine antigen or adjuvant because they enable large molecules to pass the stratum corneum via micropores, thus overcoming the limitation of the thick skin barrier in delivering the vaccine through the skin.

Based on the above-mentioned studies on gelatin-based vaccines, it is suggested that gelatin has great potential in the development of MN vaccines where it is known as having several advantages such as being less invasive than traditional injections, easy to self-administer, and not requiring cold storage [63]. This is essentially important, especially for the distribution of vaccines such as for malaria and TB vaccines, where the prevalence is usually daunting in poor countries with poor healthcare facilities. This might shift the vaccination program landscape in poor countries in the future. A summary of gelatin-based polymers for vaccine delivery and as adjuvants is presented in Table 3.

### 2.3. Albumin

**Figure 2:**
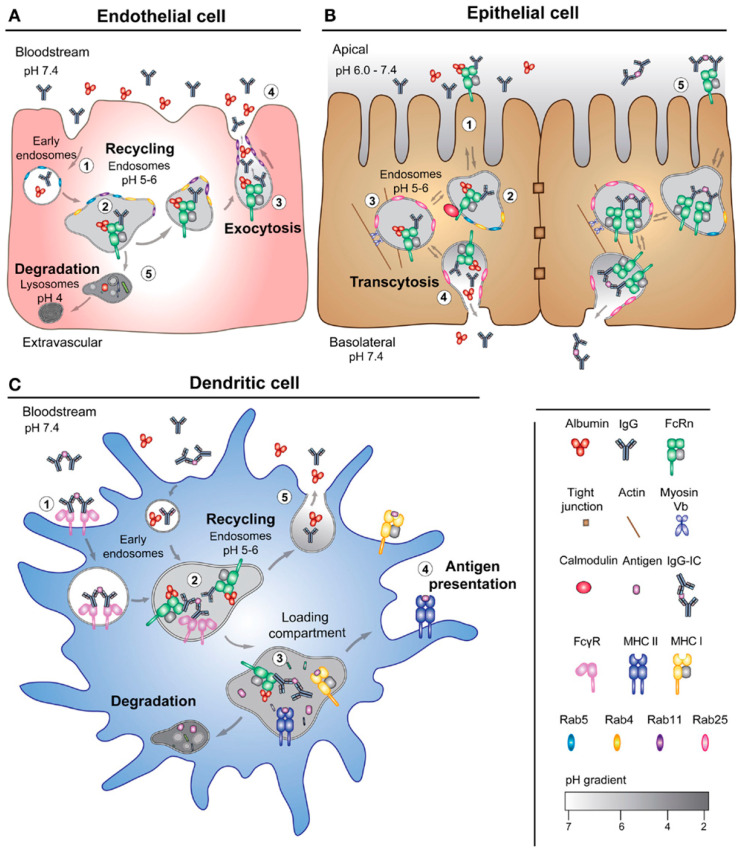
FcRn-mediated transport pathways. Adapted from Sand et al. [64].

Albumin is a highly abundant serum protein in the bloodstream similar to IgG, accounting for about 80–90% of the overall protein pool. Albumin has been recognized to be a potential polymeric biomaterial that has serum stability and longevity due to its extraordinarily extended serum half-life, which amounted to 19 to 21 days in humans [64]. Albumin binds to the neonatal Fc receptor (FcRn), which has broad biodistribution in many types of cells such as endothelial, epithelial, and DCs, as shown in Figure 2. “Albumin trafficking” enables active endosomal escape and lymph node drainage to elicit various immune signaling cascades through FcRn-mediated transport [65]. This mechanism leads to many developing therapeutic vaccines benefiting from albumin’s immunomodulatory properties.

Several studies on albumin as a nanocarrier were taken advantage of through albumin trafficking to increase vaccine efficiency through the activation of APCs. Matrix-2 protein virus-like particle (M2e VLP), an influenza ectodomain, was loaded in bovine serum albumin (BSA) MPs and showed enhanced stimulation of APCs and a further increase in APCs and M2e-specific IgG antibodies in a combination of adjuvant Alhydrogel^®^ and monophosphoryl lipid-A (MPL-A^®^) high levels in vivo [66]. Therefore, this system can potentially improve the influenza vaccine. A vaccine targeting *P. aeruginosa* PA14 strain infection using BSA-NPs containing *P. aeruginosa* ATCC 27853 antigens was used in a murine model [67]. The subjects incorporated with this system showed a high clearance of bacteria in the lungs, indicating a promising window for *P. aeruginosa* vaccines using albumin-based NPs.

The efficacy of *Pseudomonas aeruginosa* antigens in BSA-NPs was assessed after administration in vivo upon microbial infection. Macrophagic RAW 264.7 and BHK-21 cells uptake the NPs with no elicit cytotoxicity [68]. Histology assessment on a tissue section of mice treated with BSA-NPs resulted in improved skin conditions by enhancing the thickness of the skin, eliciting follicular hypertrophy, vascular crowding, and significant collagenases as well significant cellular infiltration. Moreover, BSA-NPs encapsulating rNS1 (i.e., recombinant non-structural protein 1) from Dengue virus 1 in mice presented an elevated seroconversion rate compared to rNS1 without BSA-NP immunized subjects, suggesting a good achievement in incorporating this vaccine into BSA-NPs [68]. Transdermal immunization of the measles vaccine benefited through a novel ablative laser was formulated into BSA microparticles (MPs) [69], where this novel system showed profoundly higher proliferation of MHC I/II along with CD80 and CD40, thus implying a higher immunization response was achieved by this system compared to the control group.

Albumin has also been incorporated into vaccines for cancer. The anti-tumor effect on HPV-induced cervical cancer was evaluated using NPs coated with human serum albumin (HSA) loaded with a modified and positively charged specific epitope of HPV16 E7 MHC-I followed in significantly higher E7-specific IL-10, IFN-γ, and CTL responses [70]. A stimuli-sensitive vaccine delivery system aiming to produce more targeted therapy to a target site through photothermal, near-infrared irradiation (NIR) was accomplished by encapsulating hydrophilic tumor vaccine peptide into HAS-gold NPs [71]. Both studies in vitro and in vivo showed augmentation of the anti-tumor immune response and achievable tumor ablation. Lastly, the addition of albumin to IFNβ adjuvant expands its short half-life, and its co-administration with OVA or HPV E7 long peptides heightens the specific immunity of CD8 +T cells. A profound anti-tumor effect was observed in a TC-1 tumor model [72].

In short, utilizing “albumin trafficking” through FcRn-mediated to target epithelial cells has shown a promising tool for common transdermal administration of vaccines harnessing the complexity of epithelial immunity that acts as the first-line defense in innate immunity and is key to be linked with adaptive immunity, thus enhancing the therapeutic efficacy of albumin as a vaccine carrier. A summary of albumin-based polymers for vaccine delivery and as adjuvant is presented in Table 4.

### 2.4. Hyaluronic Acid (HA)

The natural mucopolysaccharide of hyaluronic acid (HA) is a long chain of sugar molecules that are abundant in the body such as in mucus or joint fluid. This polymer is characterized as an anionic comprising disaccharides of D-glucuronic acid and N-acetyl-D-glucosamine that are connected by glycosidic bonds of β (1, 4) and β (1, 3). HA has specific CD44 receptor-mediated targeting, which is usually found on the cell-surface membrane-bound proteins of various cancer cells [73,74]. CD44 is vital in integrating cellular microenvironment cues with generating various gene expressions such as cell survival, which is crucial in vaccine cancer platforms. Cancer vaccine targeting CD44 biomarkers by using HA shows a therapeutic window for life-threatening cancer, where it serves as a critical player in self-restoration, tumor induction, metastasis, and chemoradioresistance [75]. Several studies on cancer vaccines loaded in HA were designed to target CD44. 

Exploiting the ability of HA to reprogram tumor-associated macrophages (TAM) transfection of miR-125b as antigen into HA-poly(ethylenimine)-NPs to target CD44 able to drive TAM to lung tissues with increasing (>6-fold) M1 and M2 macrophages were observed in the administered mouse model in comparison to the untreated control group [76]. This indicates the capability of HA in reprogramming TAM for vaccine anticancer therapy. However, tumor vaccines have been associated with poor immunogenicity and high heterogeneity that lowers clinical efficacy. A study was conducted to overcome this drawback by using stimuli-responsive NIR light, which was a good approach. HA-functionalized polydopamine NPs in combination with Imiquimod as adjuvant and doxorubicin (DOX) were prepared into a thermal-sensitive hydrogel [77]. Taken together, this system showed potential in theranostic tumor vaccine through prolonged retention in the tumor site along with the maturation of DC, CTL, and memory T cells in lymph nodes and spleen. 

**Figure 3:**
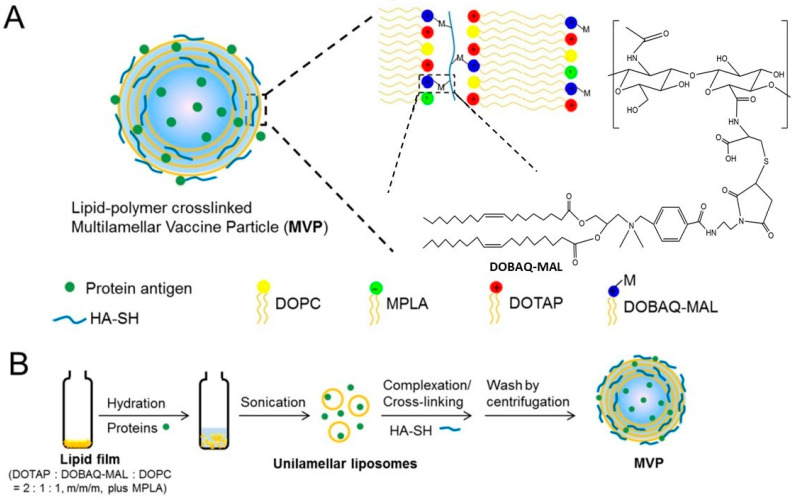
(**A**) Multilamellar vaccine particles (MVPs) system; (**B**) process of MVP synthesis. Adapted from Fan et al. [79].

In a separate study, tumor vaccine limitation was improved by loading implantable blood clots into liposome-protamine-HA NPs (LPH NPs) carrying LPH-vaccine and in LPH NPs containing siRNA (LPH-siRNA) aiming to synergistically recruit and activate DCs [78]. Remarkably, LPH-siRNA that specifically targets programmed death-ligand 1 (PD-L1), mucin-containing molecules 3, and T-cell immunoglobulin act to reduce immunosuppressor effect on DCs that leads to increased T-cell priming that tailored with tumor therapy strategy. Figure 3 shows a multilamellar vaccine particle (MVs) system comprising lipid–HA multi-cross-linked hybrid NPs for a vaccine against the Ebola virus [79]. This vaccine system showed efficacy in an 80% protection rate by inducing the immune response of CD8^+^ and CD4^+^ T cells and humoral immunity.

Next, micelles comprising HA successfully carrying OVA antigen and CpG-DNA adjuvant in the nasal vaccine were able to induce MHC II in the bone marrow DCs of a mouse to produce IgG in the blood. Indicating an active immune cascade achieved by this system [80]. HA nanocapsules were made to carry OVA antigen in an ex vivo study [81]. The interaction of immune responses was presented through the activation of macrophage, and the ability of this nanocapsule to retain OVA represents an interesting approach in needle-free vaccination.

HA has also been studied as an adjuvant in vaccine formulation in two separate studies. HA-glycine cholesterol conjugate was developed as an excipient for OVA antigen, and the antigen-specific immune response was observed in a mouse model [82]. In addition, wide T-cell-mediated immunity was activated such as CTL activation, cytokines proliferation, and induction of IgG antibodies, showing the capability of HA-derived conjugate in vaccine formulation. Lastly, modified-HA tetraglycine-l-octaarginine to be used as a mucosal vaccine adjuvant for HINI vaccines on A/Puerto Rico/8/34 (PR8) strain represents a good move, especially in the current research toward the new emerging infectious disease COVID-19 [83]. Interestingly, this modified-HA adjuvant was able to produce cross-protective capabilities by inducing IgG and IgA with PR8, and less proliferation of PR8 resulted in no profound weight loss in mice observed in experimental groups compared to control groups.

In summary, HA-based vaccine delivery has extensively been studied in tumor vaccines owing to its capability to target the CD44 receptor that is abundant in the surface protein of tumor cells [63]. The complexity of the tumor microenvironment makes treatments of cancers challenging, over the decades we have seen the treatment of cancers using biologics and chemotherapy represents hopes for cancer patients. However, they also present with limitations such as drug resistance and tumor immunosuppressive [84]. Thus, the current landscape in cancer treatments is shifted towards cancer vaccines incorporated in natural polymers including HA, which opens windows of opportunities in the immunotherapy of solid cancers. A summary of HA-based polymers for vaccine delivery and as adjuvant is presented in Table 5.

## 3. Plant-Based Polymers

### 3.1. Cellulose

**Figure 4:**
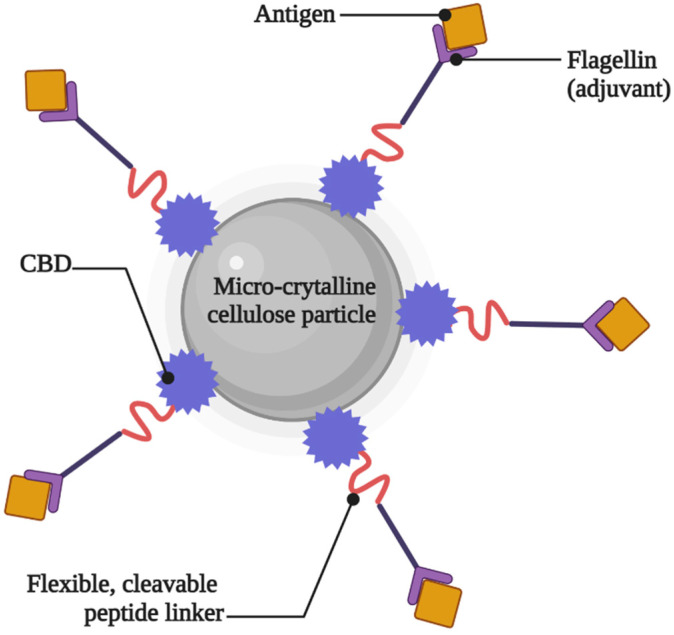
Illustration of antigen and adjuvant attached to the CBD.

Cellulose, a linear polymer composed of residual glucose, makes up the majority of plant cell walls. Due to its low solubility, stiffness, and propensity to group together to produce lengthy crystals, it is inherently resistant to biological destruction. It works well as an immunosorbent or transport material since it is resistant to chemical reactions, pharmaceutically safe, and reasonably priced. According to the literature, a variety of cellulose derivatives, including ethyl cellulose (EC), methyl cellulose (MC), hydroxypropyl cellulose (HPC), hydroxypropyl cellulose (HPMC), and ethyl hydroxy ethylcellulose (EHEC), have been used in vaccine delivery applications [85,86,87,88]. Moreover, vaccine carriers often employ microcrystalline cellulose (MCC). For example, Wang and Roman [89] formulated an MCC-based oral vaccine against the influenza virus. The cellulose-binding domain (CBD) was used in this investigation to attach a mixture of recombinant antigens, adjuvants, and targeting agents for the mucosal immune system’s inductive sites to the carrier. Meanwhile, Jeon et al. [90] used MCC as an injected vaccine carrier against erysipelas. In particular, the CBD was fused to the surface-protective protein SpaA from the Gram-positive pathogen Erysipelothrix rhusiopathiae. An illustration of the antigen/adjuvant attached to the CBD is shown in Figure 4.

A cellulose-based polymer was also studied by Mieda et al. [91] for the administration of an influenza vaccine via nasal passages. In this investigation, hydroxypropyl cellulose and sucrose were co-spray-dried to create a vaccination powder that included influenza antigen. The findings showed that the IgA antibody production was effectively stimulated in the mucosa, thereby providing advantages for infection protection. Another new vaccine carrier was designed by Ma et al. [92] from the incorporation of cellulose nanofibers and polyethyleneimine (CNF-PEI). The CNF-PEI was coupled with ovalbumin (OVA) and administered by injection for a long-term humoral immune response. The authors highlighted that the CNF-PEI has a very high potential for application in eliciting an efficient humoral immune response and avoiding the pandemic virus. However, injectable vaccinations have a number of limitations, including discomfort and inefficiency in mucosal immunity. Therefore, Chen et al. [93] examined the possibility of pH-responsive bacterial nanocellulose/polyacrylic acid (BNC/PAA) hydrogel microparticles (MPs) as carriers for oral vaccination. An in vitro entrapment efficiency and release research revealed up to 72% of OVA to be trapped in the hydrogel; the pH-dependent release of loaded OVA was also shown. OVA and cholera toxin B (CTB) were used as model antigens and mucosal adjuvants in in vivo oral vaccination. The in vivo immunization showed that animals given OVA and CTB-loaded hydrogel MPs orally produced much larger levels of serum anti-OVA IgG and mucosal anti-OVA IgA in the intestinal washes than mice given OVA intramuscularly. Lee et al. [94] also investigated the pH-sensitive and mucoadhesive polymeric MPs, but they used a double-emulsion approach using cellulose acetate phthalate (CAP) to boost the immunological response to the FMDV subunit vaccine. CAP thiolation enhances CAP’s mucoadhesive properties to extend MPs transit time through the digestive system. Due to its pH sensitivity, thiolated CAP (T-CAP) slowed down the release of antigens in the acidic pH of the stomach but released more antigens in the neutral pH of the small intestine. When administered orally, a chimerical multi-epitope recombinant protein serving as the FMD subunit vaccine delivered by T-CAP MPs successfully stimulated a mucosal IgA response in Peyer’s patches.

Moreover, Song et al. [95] reported the synthesis of cellulose-based NPs in aqueous media using the oppositely charged materials carboxymethyl cellulose (CMC) and quaternized cellulose (QC). CMC–QC NPs were first loaded with pEGFP-N1, a plasmid DNA encoding the enhanced green fluorescent protein (EGFP), as a model in COS-7 cells. Then, the CMC–QC NPs were loaded with a DNA vaccine, pMASIA-tPAs-tE2.2, to defend against the virus that causes bovine viral diarrhea (BVDV). The findings showed that CMC–QC NPs could bind DNA effectively and that optimized DNA-loaded NPs could induce transfection in COS-7 cells with remarkable efficacy.

Additionally, recent research showed that cellulose-based substances may be utilized in vaccine formulations as adjuvants for proteins, antigens, or DNA in the form of particles, nanowires, or nanofibers to improve immune response by boosting the release of pro-inflammatory cytokines [96,97]. In an animal vaccination experiment, in order to prove that nanocellulose materials can increase the IgG response to OVA in a mouse model, Wang et al. [97] demonstrated the potent impact of cellulose nanocrystals (CNCs) and nanofibers (CNFs) on the maturation of bone-marrow-derived dendritic cells (BMDCs) and the production of IL-1. The findings obtained presented that all CNFs and CNCs had a considerable ability to increase the antiOVA IgG response. Roohani et al. [98] synthesized new green nanocomposites based on poly(vinyl alcohol) (PVA) with CNCs and functional Fe_3_O_4_ (FMNP) as a potential adjuvant. Despite the fact that PVA has been utilized widely in the biomedical area, including the administration of drugs and vaccines, its usage has certain limitations, including poor heat stability, high flammability, and only moderately strong mechanical qualities. The incorporation of FMNP as a compatibilizing agent along with an adjuvant of CNCs resulted in an improvement in its storage modulus and thermal stability, particularly at high temperatures, thus expanding its uses.

In brief, cellulose helps transport vaccines and adjuvants to the target region, followed by regulated release, which increases the immune response. Interestingly, the cellulose carrier was discovered to have been broken down by the cell after delivery. The main interest in the cellulose-based vaccine is its robust applications for oral vaccines, which exhibit advantages over parenteral vaccines, targeting various intestinal immune responses at the systemic and mucosal levels. Oral vaccination is also responsible for inducing protective secretory IgA responses that synergistically work with systemic immunity to provide protection wholly. Although the harsh environment of the gastrointestinal tract with low pH and intestinal proteases makes oral vaccination challenging, cellulose derivatives have potentially produced protection for oral vaccines, which may contribute to the development of the next generation of oral subunit vaccines. A summary of cellulose-based polymers for vaccine delivery and as adjuvants is presented in Table 6.

### 3.2. Alginate

**Figure 5:**
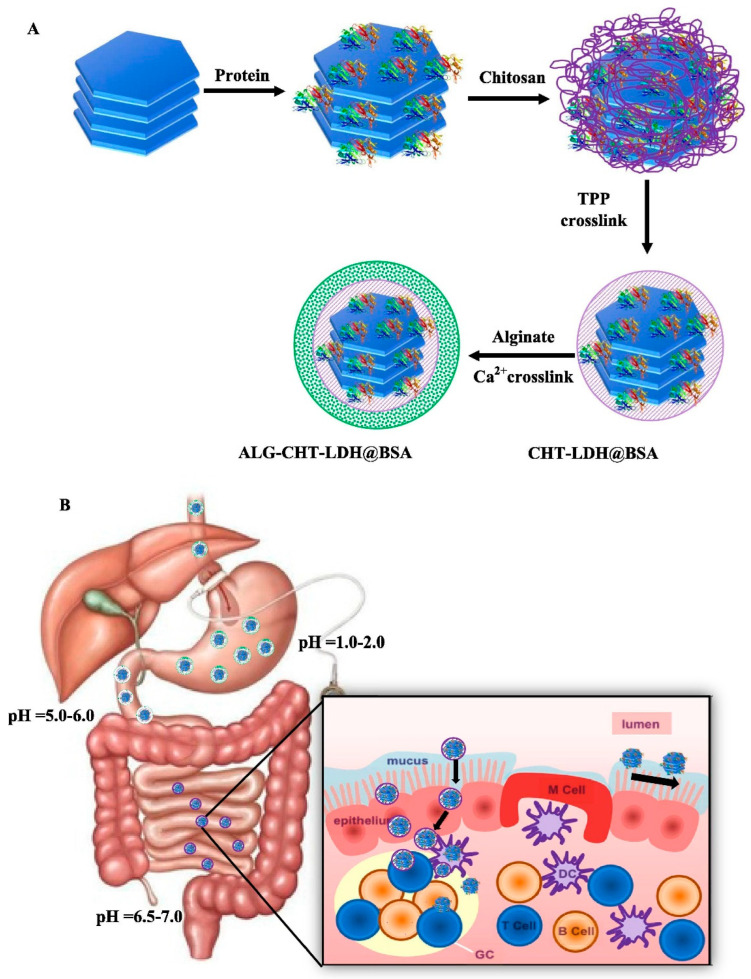
Schematic illustration of (**A**) the synthesis steps and (**B**) the action mechanism of ALG-CHT-LDH NPs. Adapted from Yu et al. [99].

Alginate, which is composed of (1–4) connected D mannuronic acid and L guluronic acid, is found in the cell walls of brown sea algae. Alginate has been widely utilized for vaccine administration since it may prolong antigen release and increase immunogenicity more than conventional vaccinations. Moreover, it may lessen deterioration in an acidic environment during nasal and oral delivery due to its mucoadhesive characteristics. Yu et al. [99] investigated the application of layered double hydroxide NPs (ALG-CHT-LDH) coated with alginate and chitosan polymers as a pH-sensitive and mucoadhesive polymer for oral vaccination administration. As presented in Figure 5, ALG-CHT-LDH nanocomposites may safely transit through the stomach while carrying protein antigen (BSA) to protect CHT-LDH@BSA from acidic destruction (pH 1.2), and the alginate separates from ALG-CHTLDH@BSA after traversing the stomach. According to Biswas et al. [100], alginate coating may successfully preserve an antigen in an acidic environment for at least two hours. The capacity to preserve antigens in the gastrointestinal environment, delayed release kinetics, systemic and mucosal immune responses, and low cytotoxicity of chitosan NPs coated with sodium alginate suggest that they may be a good platform for oral vaccination delivery. Another study by Mosafer et al. [101] demonstrated that alginate improves the stability and alters the immunostimulatory characteristics of chitosan and trimethyl chitosan NPs. Therefore, the trimethyl chitosan–alginate formulation may be thought of as a successful intranasal antigen delivery strategy for nasal vaccinations. Additionally, a mice model was used to assess vaccination surface antigens that had been adjuvanted with chitosan, alginate, or both [102]. Then, the responses were compared to control animals that received no adjuvant and conventional alum adjuvant. The findings demonstrated that the alginate-/chitosan-based vaccination produced a more potent immune response than conventional alum-adjuvant and without adjuvant. In light of this, the alginate-based system revealed promising results for the administration of the vaccine in nasal and oral mucosal immunization. Alginate is another polymer with natural mucoadhesive properties, and the functional groups on its chemical structure are responsible to induce various interactions by attaching to mucus in the oral cavity, which contributes to its application in both oral and mucosal vaccines. A summary of alginate-based polymers for vaccine delivery and as adjuvants is presented in Table 7.

## 4. Microbe-Based Polymers

### 4.1. Dextran

**Figure 6:**
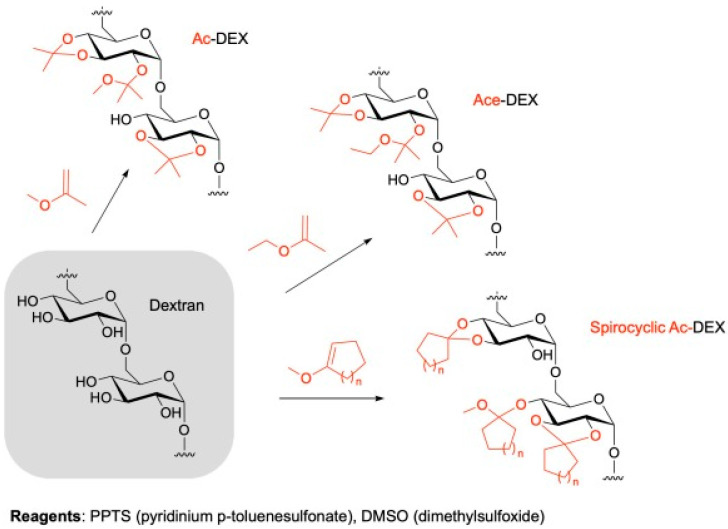
Strategies for the acetylation of dextran. Adapted from Prasher et al. [105].

Dextran is a microbe-based natural polymer mainly isolated from Gram-positive bacteria such as facultatively anaerobic cocci from strains Leuconostoc and Streptococcus [103]. Dextran has high solubility in water due to its neutral complex amylopectin-chain glucan comprising α-1,6-glycosidic linkages in the middle of glucose monomers [104]. The high solubility properties of dextran make it unfavorable in MP production. Thus, dextran has been modified into acetylated dextran-Ace-DEX through a single-stage acetyl formation with 2-methoxypropene to produce an insoluble modified biopolymer. Figure 6 shows the strategies of dextran acetylation. As discussed in the next section, insoluble Ace-DEX is used to design MPs and has been used in many vaccine formulations. Ace-DEX in vaccine delivery is said to be either fast or slow-degrading (i.e., controlled based on polymer cyclic acetyl coverage) polymer that can exhibit great innate immunity stimulation through the tunable and sustained release of antigen and adjuvant.

Ace-DEX MPs were used to formulate an influenza vaccine using antigen matrix protein 2 (M2e) [106] and hemagglutinin antigen (HA) [107]. Both formulations elicited great protection against the influenza virus by cross-reactivity [106] and sustained release of adjuvant CpG in a combination of HA antigens [107]. In addition, Ace-DEX MPs encompassing a potent immunostimulatory adjuvant stimulator of interferon genes (STING) agonist cyclic GMP-AMP (cGAMP) showed better efficacy conducted by this adjuvant [108,109]. This formulation resulted in more than 10 times dose-sparing outcomes in contrast to other studies, indicating a promising window of opportunity in influenza vaccines and COVID-19 vaccines, which currently undergo limitations such as waning immunity [108]. Plus, cGAMP in combination with TLR7/8 agonist resiquimod (R848) in Ace-DEX showed a greater cellular immune response and fair Th1/Th2 humoral response compared to single-activity cGAMP Ace-DEX MPs and PAMPs carried in different MPs [109].

Ace-DEX MPs are also used to encapsulate OVA or murabutide as a model antigen or adjuvant, respectively [110]. Controlled adjuvant delivery exhibited humoral and cellular responses that are earlier and later in fast-degrading MPs and slow-degrading MPs, respectively, whereas fast-degrading MPs produced greater antibodies and secretion of cytokine in controlling antigen delivery compared to slow-degrading MPs. Thus, flexible control can be achieved by Ace-DEX, which serves as a great tool in vaccine delivery. A new formulation of vaccine malaria was investigated in merozoite surface protein 2 (MSP2) antigen using Ace-DEX MPs as a vaccine excipient and alum as an adjuvant [111]. This formulation was demonstrated in a promising malaria vaccine via Th1-biased response, indicating high anti-inflammatory responses towards malaria infection.

The much-anticipated type of dextran is Ace-DEX, where there are many studies of using Ace-DEX NPs in delivering many types of vaccines Ace-DEX in vaccine delivery due to the capability of Ace-DEX NPs that can be modified its polymer cyclic acetyl coverage to achieve fast- or slow-degrading polymer importance in the sustained release of antigen or adjuvants. Table 8 presents a summary of dextran-based polymers for vaccine delivery and as adjuvants.

### 4.2. β-Glucan

β-glucan is the most plentiful glucose polysaccharide inside the cell wall of fungi and yeast such as *Saccharomyces cerevisiae.* There is a wide variety of β-glucan depending on the carbon bonds such as (1→3), (1→4), and (1→6) in the D-glucose units [112]. The immunomodulatory effects possessed by β-glucan known by trained immunity induction provide great potential for β-glucan as a vaccine adjuvant [113]. Trained immunity is a de facto innate immune memory elicited through adaptive immune response such as tissue-resident memory (TRM) and epigenetic reprogramming of transcriptional pathways [114]. Exploiting β-glucan receptors such as Dectin-1, lactosylceramide, CR3, selected scavenger receptors, and the TLR on immune cells facilitates the therapeutic efficacy of vaccines delivered when incorporated with β-glucan [115]. These various receptors β-glucan are vastly important in trained immunity that can be further applied in vaccine formulation, especially for the current new emerging infectious disease COVID-19 that possesses waning immunity.

**Figure 7:**
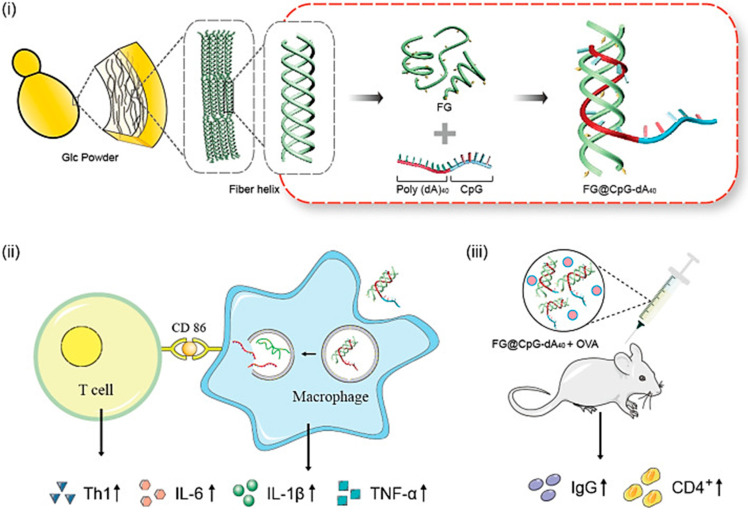
Development process and immunotherapeutic results of FG@CpG-dA_40_. Adapted from Xu et al. [117].

One unique property of β-glucan compared to other polymer is it usually exist in triple-helix configuration including LNT from *Lentinus edodes*, SPG from *Schizophyllum commune*, CUR from *Alcaligenes faecalis*, Sclg from *Sclerotium glucanicum*, and many others [116]. Chemical modification, high temperature or pH alteration, and dimethyl sulfoxide (DMSO) solution can be used to obtain single chains from the unwinding process of triple-helix *β*-glucan. This allows *β*-glucan to wrap up nearby guest molecules such as DNAs/RNAs, which showed promising capability in vaccine formulation. β-glucan can serve both as a carrier and adjuvant at the same time in vaccine formulation. In a recent study, high wrapping capability is shown by a hybrid helix formed by single-helical formylated β-glucan with DNA CpG-poly(dA) to form FG@CpG-dA40, resulting in the upregulation of the inflammatory factors at the mRNA and protein levels and the production of an immune-boosting consequence on mice vaccinated with OVA, as shown in Figure 7 [117].

A novel anticancer vaccine candidate was designed by conjugating β-glucan to a MUC1 peptide antigen, which allowed delivering and binding of antigen to myeloid cells [118]. This simultaneously triggers immune activation such as profound cross-reactivity with the tumor cell line of MCF-7 shown by superior anti-MUC1 antibody titers and raises IL-6 and IFN-g levels in mice sera.

On the other hand, the delivery of hepatitis B surface antigen (HBsAg) was studied in two separate studies using β-glucan particles plus aluminum salt [119] or PDMAEMA: PβAE/DNA polyplexes [120]. β-glucan-aluminum salt particles were designed to carry hepatitis B surface antigen (HBsAg), which presented with a higher serum anti-HBsAg IgG titer in WT and HBV-Tg mice compared to administration of antigen only [119]. Enhanced polyplexes transfection activity and greater luciferase gene expression were shown in the existence of β-glucan particles compared to the control [120]. Both studies presented a safe and promising system for HBsAg antigen delivery by using β-glucan particles. In a recent phase II clinical trial, oral β-glucan was administered together with the GD2/GD3 vaccine for the treatment of high-risk neuroblastoma [121]. Adjuvant effects of β-glucan on three single nucleotide polymorphisms (SNPs) of dectin-1 were correlated with the high anti-GD2-IgG1 titer and overall improvement in patients’ survival, providing great promise of β-glucan as a vaccine adjuvant.

In summary, trained immunity induction by β-glucan through various recognition receptors on immune cells has led to much successful research on β-glucan as a vaccine adjuvant, and it is currently proposed as an adjuvant and carrier for COVID-19 vaccines, as discussed in the next section. Trained immunity is of much importance to reducing dose dependency, as shown by various vaccination programs and for COVID-19 vaccination. This dose dependency could be hindered by harnessing the ability of β-glucan to reprogram the epigenetic and enhance vaccine responsiveness. Table 9 presents a summary of β-glucan-based polymers for vaccine delivery and as adjuvants.

## 5. Novel Natural Polymeric Applications in COVID-19 Vaccine Formulation

Researching the various applications of natural polymers targeting influenza viruses gives insights to researchers using the system to enhance COVID-19 vaccine formulation. Severe acute respiratory syndrome coronavirus 2 (SARS-CoV-2) enters the host through the respiratory tract (i.e., nose and other mucosae) by binding its viral spike protein (Sp) to the cell-surface receptor angiotensin-converting enzyme 2 (ACE2). Targeting SARS-CoV-2 viruses through mucosal vaccine delivery has shown promising possibilities compared to parenteral vaccination. Chitosan has been applied in various applications of the mucosal vaccine, as shown in Table 1. Chitosan is advantageous due to its mucoadhesion properties, which exhibit positive charges, and negatively charged spike proteins, which have led to many discoveries of COVID-19 vaccine-based chitosan [122,123].

Targeting SARS-CoV-2 viruses through mucosal vaccine delivery has shown promising possibilities compared to parenteral vaccination. A study on inhalable nanovaccines encompassing SARS-CoV-2 Sp into chitosan strategized to induce potent spike-specific antibody immune response in bronchoalveolar lavage and lungs [122]. SARS-CoV-2 infectivity is attributable to the ACE receptor’s high expression on the membrane surface of respiratory epithelial cells [124]. Thus, the nasal formulation of the COVID-19 vaccine aims to improve nasal moisture and produce an ACE2 receptor inhibitor. Chitosan–hydrogel comprising an ACE2 receptor inhibitor was designed and resulted in a good formulation of active pharmaceutical ingredients (APIs) required to achieve the inhibitory concentration (*c* ≥ IC_50_) [125].

A study suggested an inhaled chitosan-coated DNA vaccine coding a secreted spike protein portion of COVID-19 responsible for blocking the ACE2 receptor site [126] by triggering both MHC I and MHC II with limited systemic side effects and producing humoral and cytotoxic immunity. In a recent in vivo study, NARUVAX-C19/Nano, an intranasal subunit vaccine, was enclosed in mannose-conjugated chitosan NPs using the SARS-CoV-2 Sp receptor-binding domain (RBD) as an antigen and CpG55.2 adjuvant [127]. Two times IgA antibody secretion and significant Th1 responses were achieved by the BALB/c mice NP vaccines group compared to the intramuscular alum-adjuvanted RBD vaccine group. Moreover, protection against wild-type SARS-CoV-2 (D614G) was exhibited by Syrian hamsters administered the same NP formulations through non-significant weight loss and decreased viral load in the lungs. Injectable hydrogels formulated using chitosan and glycerophosphate were used to overcome the rapid degradation of adjuvant and epitope cowpea mosaic virus (CPMV) for COVID-19 [128]. The results showed a slow and sustained release (over 20 weeks) of cyanine 5-CPMV particles in vitro and in vivo with improved antibody titers in hydrogel than soluble vaccine candidates.

On the other hand, chitosan was used as a vaccine adjuvant in MN target SARS-CoV-2 nucleocapsid protein [129] and RBD polypeptides vaccine for COVID-19 [130]. The MN-based vaccines resulted in a balanced ratio of IgG1 and IgG2A nucleocapsid antibodies [129]. The RBD polypeptide vaccine intracutaneously administered in mice produced RBD-specific antibodies, which was sustained for five months [130]. Cross-reactivity was also observed against RBD antibodies with various SARS-CoV-2 variants (i.e., alpha, beta, and delta variants). Intranasally administered vaccines also elicited RBD-specific mucosal IgA antibodies key elements for COVID-19.

**Figure 8:**
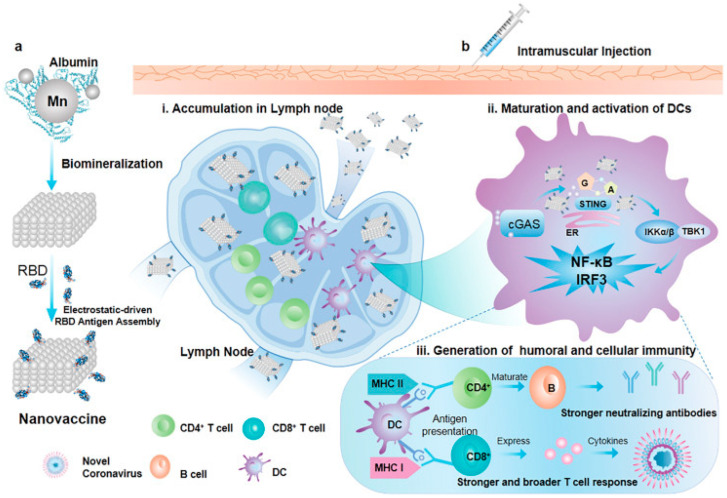
Mechanism of action elicited by new albumin-based nanovaccine targeting coronavirus to induce humoral and cellular responses. (**a**) Schematic illustration of the construction of MnARK and the MnARK nanovaccine. (**b**) Schematic representation of the utilization of the nanovaccine for protection from novel coronavirus infection. Adapted from Wang et al. [131].

Fascinated by the ability of albumin to encourage lymph node drainage in improving vaccine effectiveness, an albumin nanovaccine was developed using a manganese nanoadjuvant (MnARK) and the RBD of Sp [131]. This MnARK vaccine showed greater anti-virus neutralizing effects in vitro against pseudovirus approximately 270-fold and more than 8-fold on live coronavirus than the alum-adsorbed RBD vaccine. This albumin-based nanovaccine is also effective in simultaneously delivering RBD and MnARK antigens to lymph nodes, boosting cellular internalization and the stimulation of immune cells (i.e., DCs, CD4+, and CD8+ T lymphocytes). Figure 8 shows the mechanism of action elicited by the new albumin-based nanovaccine targeting coronavirus to provoke humoral and cellular responses. The rationale of this study may provide new insights into the COVID-19 vaccination strategy.

In addition, Kim et al. [132] designed a microneedle-based vaccine delivery system using carboxymethyl cellulose at room temperature for the SARS-CoV2 antigenic protein, which may one day prove to be a novel vaccine delivery method for SARS-CoV2 vaccination. A smart mushroom-inspired imprintable and lightly detachable (MILD) MN system for the COVID-19 vaccine was developed using alginate and CMC [133]. This type of vaccine does not require cold-chain equipment, which is important for large-scale vaccination programs. The MILD system that both serves as vaccine delivery and in situ data storage produced a superior level of antibodies against the SARS-CoV-2 RBD in vivo with no systemic toxicity and local damage elicited after loading inactivated SARS-CoV-2 virus-based vaccines. In this study, a three-stage MN array manufacturing approach was used to create dissolvable MN arrays containing the protein from CMC [134,135]. Strong and persistent antigen-specific antibody responses were produced when MERS-S1 subunit vaccinations were administered by MN arrays. Then, the MN arrays delivered SARS-CoV-2 S1 subunit vaccines induced strong antigen-specific antibody responses that were visible 2 weeks after vaccination.

A microbe-based polymer, dextran, has also been used in designing a carrier for the COVID-19 vaccine by incorporating SARS-CoV-2 and R837 CTL epitope to partially oxidized Ace-DEX NPs [136]. Robust activities of CTL were observed towards SARS-CoV-2 through several CTL epitopes, two Sp of SARS-CoV-2 (Sp1 and Sp2) and one each of Sp9 and Sp11 from ORF1ab polyprotein and nucleocapsid (N) protein, respectively. Together with R837 and these four epitopes, this study highlighted an excellent translation possibility against SARS-CoV-2. A hopeful vaccine delivery for COVID-19 was also shown by β-glucan particles to carry the antigen SARS-CoV-2 WA1 strain RBD against SARS-CoV-2 variants (i.e., Omicron, Delta, and Gamma) in vivo where it elicits durable humoral and cellular immune responses, presenting a therapeutic window in formulating COVID-19 [137]. Moreover, the potential of β-glucan as an adjuvant for SARS-CoV-2 has been widely discussed as harnessing β-glucan abilities in eliciting various immune cascades through β-glucan recognition receptors on various immune cells and trained immunity [113,115,138].

## 6. Challenges and Future Perspectives

Natural polymeric delivery vehicles have the potential to be reliable and secure carriers for next-generation vaccinations because they have the ability to act as their own adjuvants and elicit the proper immune responses. However, the reported studies did not always comprehensively evaluate and compare the advantages of polymeric systems with commonly used standard adjuvants, where currently only a few have been approved. Challenges of using natural polymers such as the risk of anaphylaxis reactions in gelatin-based vaccines, especially in patients with alpha-gal-sensitization. Alpha-gal syndrome induces IgE sensitization [139] towards carbohydrate galactose-α-1,3-galactose (alpha-gal), usually observed in gelatin products [140]. Gelatin-encompassing vaccines for diseases such as zoster, varicella, mumps, measles, and rubella in patients who possess alpha-gal syndrome showed positive basophil activation test (BAT) (i.e., indicates possible anaphylaxis reaction) [141]. Gelatin-based vaccines for diphtheria, tetanus toxoids [142,143], measles, yellow fever, and varicella [144] caused urticaria and angioedema, as well as anaphylaxis. Therefore, careful monitoring must be conducted in patients administered gelatin-based vaccines. Natural polymer-based vaccines must undergo clinical translation, which requires strict regulation before they can be used for patients’ treatment, and long-term monitoring of the toxicity of natural-polymer-based nanovaccines must be conducted. Moreover, the challenge is to produce natural-polymer-based vaccines that elicit greater immunizations that can reduce multiple doses of vaccinations that are often related to greater side effects and toxicity.

Thus, further research is directed at manipulating the ability of natural polymers in trained immunity. There is much more space to explore the long-term functional programming of innate immune cells by natural-polymer-based adjuvants other than β-glucan. Trained immunity is foreseen as a promising strategy to control COVID-19-like pandemics, where current COVID-19 vaccines require multiple boosters. In addition, cancer vaccines incorporated in natural polymers are much anticipated to be translated as a treatment modality in oncology to overcome tumor immunosuppressive and drug resistance, which may also be paving the way to the future of personalized vaccines for precision medicine.

## 7. Conclusions

A good vaccine administration strategy not only ensures that the vaccine is delivered properly but also contributes significantly to inducing a potent immunological response in the immune systems of both humans and animals. Therefore, the establishment of a vaccine delivery system is essential for the vaccine’s effectiveness by increasing its impact and promoting an immunogenic response. The use of polymer-based technologies for vaccine delivery has been hailed as an innovative strategy. For effective vaccine distribution, several formulations including injectable delivery systems, injectable hydrogel-based biobullets, microspheres, and microneedles have been developed. These delivery mechanisms are important in the immunological response mediated by antigens. According to reports, these methods have proved effective in delivering the cargo vaccine or antigen molecule through the nasal or injectable route and may trigger a powerful immune response. Particularly, the advancement of natural polymers such as chitosan, gelatin, albumin, HA, cellulose, alginate, dextran, and β-glucan in the creation of composites based on natural polymers for vaccine delivery systems has been described. Due to the biocompatibility of these polymer composites, vaccine delivery experiments have shown encouraging results.

## Figures and Tables

**Table 1:** Novel studies on mucosal vaccine delivery using chitosan-based-delivery vehicles.

Formulation	Application	Vaccine Antigen	Immune Response	Ref.
N-trimethyl chitosan (TMC) NPs	Influenza	Influenza M2 protein	Enhanced M2e-specific IgG antibody and BALF anti-M2e IgA levels in the serum Th1-biased immune response	[38]
Chitosan–hydrogel	Influenza	Ovalbumin antigen	CD8^+^ T cells activation of Trm in the nasal mucosa.	[39]
*N*-2-hydroxypropyl trimethyl ammonium chloride chitosan/*N*,*O*-carboxymethyl chitosan NPs	n/a	Bovine serum albumin	Production of lymphocytes and pro-inflammatory factors	[40]
2-hydroxypropyltrimethyl ammonium chloride chitosan-based hydrogel	H5N1 influenza A	H5N1 antigens	Sustained antigen release time in the nasal cavity Strong systemic responses through IgG, IgG1, IgG2a, and HI production	[41]
Chitosan MPs cross-linked with glyceraldehyde	Leishmania	p36/LACK leishmanial antigen (LACK-DNA)	Increased proliferation and IFN-g and decreased IL-10 production	[42]
Modified chitosan microspheres loaded with P6 mannose	Nontypeable *Haemophilus influenzae* (NTHi) infection	NTHi outer membrane protein P6	Mixed Th1/Th2/Th17-type immunity Promoting lymphocyte proliferation Induce MHC class I/II and CTL immune responses	[43]

BALF: Bronchoalveolar lavage fluid; CTL: Cytotoxic T lymphocyte; IgG: Immunoglobulin G; M2e: extracellular domain of M2; MHC: Major histocompatibility complex; MPs: Microparticles; n/a: Not applicable; NPs: Nanoparticles; Th1/2/17: T helper 1/2/17; Trm: Tissue-resident memory T.

**Table 2:** Summary of chitosan-based polymers for vaccine delivery and as adjuvants.

Chitosan as Vaccine Delivery					
Formulation	Application	Vaccine Antigen/ Adjuvant	Immune Response	Administration Route	Ref.
Chitosan-tripolyphospha-te NPs	Cervical cancer	pDNA	Increased E7 antigen transcription	n/a	[30]
Nanosphere of chitosan/trimethyl chitosan-coated PLGA	Tuberculosis	HspX, EsxV and PPE44 *Mtb* antigens/resiquimod	Th1-dominant response	n/a	[32]
Polyacrylamide/chitosan iontophoresis-driven MN	n/a	OVA	Improved immune response compared to traditional intramuscular injection	Transdermal	[34]
**Chitosan as adjuvant**					
**Formulation**	**Application**	**Vaccine Antigen**	**Immune Response**	**Administration Route**	**Ref.**
Chitosan-modified nanographene oxide	Hepatitis E virus	P239	Induced production of IgG antibodies and activation of cytokines	n/a	[26]
Chitosan-*Salmonella Typhi* porins MPs and NPs	Toxoplasmo-sis	rGRA1 and rBAG1	Higher cellular and humoral response by MPs compared to the NPs Increasing the protection against *T. gondii* in MPs	n/a	[27]

IgG: Immunoglobulin; MPs: Microparticles; n/a: Not applicable; NPs: Nanoparticles; *Mtb: Mycobacterium Tuberculosis*; OVA: Ovalbumin; pDNA: Plasmid DNA; Th1: T helper 1.

**Table 3:** Summary of gelatin-based polymers for vaccine delivery and as adjuvants.

Gelatin as Vaccine Delivery					
Formulation	Application	Vaccine Antigen/ Adjuvant	Immune Response	Administration Route	Ref.
Gelatin-based MN	Malaria	*Plasmodium falciparum* surface protein P47/CpG	Activate the TLR9 signaling and DC	Transdermal	[56]
Aminated nanoparticulate MN-based gelatin	Tetanus toxoid	Anti-tetanus toxoid	High production of antibodies	Transdermal	[57]
Gelatin NPs	Cancer	OVA/polyinosinic: polycytidylic acid	EG7 tumor growth inhibition in C57BL/6 mice	Mucosal	[59]
Mannosylated gelatin NPs	PRRSV	Inactivated PRRSV	Inactivated PRRSV-infected cells via improved T-cell signaling cascade	n/a	[61]
Gelatin-based MN	Botulinum toxin	AHc	Good immunogenicity and protection efficacy of AHc vaccine in 6 months of storage at room temperature	Transdermal	[62]

DC: Dendritic cell; MN: Microneedle; n/a: Not applicable; NPs: Nanoparticles; OVA: Ovalbumin; PRRSV: porcine Reproductive and respiratory syndrome virus; TLR: Toll-like receptor.

**Table 4:** Summary of albumin-based polymers for vaccine delivery and as adjuvants.

Albumin as Vaccine Delivery					
Formulation	Application	Vaccine Antigen/Adjuvant	Immune Response	Administration Route	Ref.
BSA MPs	Influenza	Matrix-2 protein virus-like particle/Alhydrogel^®^ and monophosphoryl lipid-A	Enhanced stimulation of APCs	n/a	[66]
BSA-NPs	*P. aeruginosa* PA14 strain infection	*P. aeruginosa* ATCC 27853	High clearance of bacteria in the lung	n/a	[67]
BSA-NPs	Microbial skin infection	*Pseudomon-as aeruginosa* antigens	Improved skin condition	n/a	[68]
BSA-NPs	Dengue	Non-structural protein 1 from Dengue virus 1	Elevated seroconversion rate compared to rNS1 without BSA-NPs	n/a	[68]
BSA MPs	Measles	Measles antigen	High proliferation of MHC I/II plus CD80 and CD40	Transdermal	[69]
HAS NPs	HPV-induced cervical cancer	HPV16 E7 MHC-I specific epitope	High E7-specific IL-10, IFN-γ, and CTL responses	n/a	[70]
Albumin	Cancer	OVA or HPV E7 long peptides	Heighten specific CD8 +T cell immunity Anti-tumor effect was observed in TC-1 tumor model	n/a	[72]

APCs: Antigen-presenting cells; BSA: Bovine serum albumin; HPV: Human papillomavirus; MHC: Major histocompatibility; MPs: Microparticles; n/a: Not applicable; NPs: Nanoparticles; OVA: ovalbumin.

**Table 5:** Summary of HA-based polymers for vaccine delivery and as adjuvants.

HA as Vaccine Delivery					
Formulation	Application	Vaccine Antigen/Adjuvant	Immune Response	Administration Route	Ref.
HA-poly(ethylenimine) NPs	Cancer	miR-125b	Drive TAM to lung tissues with increasing M1 and M2 macrophages	n/a	[76]
HA-functionalized polydopamine NPs in the hydrogel system	Cancer	Doxorubicin/Imiquimod	Maturation of DC, CTL, and memory T cells in lymph nodes and spleen	n/a	[77]
Liposomes-protamine-HA NPs	Cancer	siRNA	Target PD-L1 and mucin-containing molecules 3	n/a	[78]
Lipid–HA multi-cross-linked hybrid NPs	Ebola	Protein	80% protection rate through inducing CD8^+^ and CD4^+^ T cell	n/a	[79]
HA-coated micelles	n/a	OVA/CpG-DNA	Induce MHC II in bone marrow DCs and produce IgG	Mucosal	[81]
**HA as Adjuvant**					
**Formulation**	**Application**	**Vaccine Antigen**	**Immune Response**	**Administration Route**	**Ref.**
HA-glycine cholesterol (HACH) conjugate	n/a	OVA	CTL, cytokines, and IgG antibodies production	n/a	[82]
Modified-HA tetraglycine-l-octaarginine	H1N1	H1N1 antigen	Cross-protective capabilities by inducing IgG and IgA with PR8 Less proliferation of PR8	n/a	[83]

CTL: Cytotoxic T lymphocytes; DC: Dendritic cell; HA: Hyaluronic acid; IgG: Immunoglobulin G; MHC: Major histocompatibility complex; MPs: Microparticles; n/a: Not applicable; NPs: Nanoparticles; OVA: Ovalbumin; PD-L1: Programmed death-ligand 1; TAM: Tumor-associated macrophages.

**Table 6:** Summary of cellulose-based polymers for vaccine delivery and as adjuvants.

Formulation	Application	Vaccine Antigen/Adjuvant	Immune Response	Administration Route	Ref.
MCC	Influenza	M2e protein/Flagellin	Enhanced IgG antibody	Oral	[89]
Hydroxypropyl cellulose and sucrose	Influenza	HA and virion	Enhanced IgG antibody	Nasal	[91]
CNF-PEI	BMDC	OVA	High long-term humoral immune response	Injection	[92]
BNC/PAA	Raji B cell and HT29-MTX-E12	OVA	Enhanced IgA and IgG antibodies	Oral	[93]
T-CAP MPs	FMDV	M5BT	Enhanced IgA antibody	Oral	[93]
CMC–QC nanoparticles	BVDV	pEGFP-N1 and pMASIA-tPAs-tE2.2	Enhanced E2 protein level	Injection	[95]
CNC-CNF	BMDC	OVA	Enhanced IgG antibody	Injection	[97]

**Table 7:** Summary of alginate-based polymers for vaccine delivery and as adjuvants.

Formulation	Application	Vaccine Antigen/Adjuvant	Immune Response	Administration Route	Ref.
ALG-CHT-LDH	Caco-2, HT-29 and Raw264.7 cells	BSA	Enhanced the uptake of protein antigen at Caco-2, HT-29, and Raw264.7	Oral	[99]
CHT-ALG	HT-29	Measles	Enhanced immunological responses	Oral	[100]
Alginate coated chitosan	Influenza	PR8	Enhanced IgG2a and IgG1 antibodies	Nasal	[101]
Alginate-chitosan	Listeriosis	Gamma-irradiated L. monocytogenes	CD3+ T-cell proliferation and enhanced memory CD4+ and CD8+ T cell	Oral	[102]

**Table 8:** Summary of dextran-based polymers for vaccine delivery and as adjuvants.

Dextran as Vaccine Delivery					
Formulation	Application	Vaccine Antigen/Adjuvant	Immune Response	Administration Route	Ref.
Ace-DEX MPs	Influenza	Antigen matrix protein 2	Great protection against the influenza virus via cross-reactivity	n/a	[106]
Ace-DEX MPs	Influenza	Hemagglutinin/CpG	Great protection against the influenza virus	n/a	[107]
Ace-DEX MPs	Influenza	cGAMP	10 times dose-sparing effects	n/a	[108]
Ace-DEX MPs	Influenza	cGAMP and TLR7/8 agonist resiquimod (R848)	Great cellular immune response and balanced Th1/Th2 humoral response	n/a	[109]
Ace-DEX MPs	n/a	OVA/murabuti-de	Great antibodies and secretion of cytokine	n/a	[110]
Ace-DEX MPs	Malaria	Merozoite surface protein 2/alum	Th1-biased response	n/a	[111]

Ace-DEX: Ecetalated dextran; cGAMP: cyclic GMP-AMP; MPs: Microparticles; n/a: Not applicable; NPs: Nanoparticles; OVA: Ovalbumin; Th: T helper; TLR: Toll-like receptor.

**Table 9:** Summary of β-glucan-based polymers for vaccine delivery and as adjuvants.

β-Glucan as Vaccine Delivery					
Formulation	Application	Vaccine Antigen/Adjuvant	Immune Response	Administration Route	Ref.
β-glucan hybrid helix	n/a	OVA/CpG-poly(dA)	Upregulating inflammatory factors at mRNA and protein levels	n/a	[117]
β-glucan conjugates	Cancer	MUC1 peptide	High anti-MUC1 antibody titers Elevated IL-6 and IFN-g levels	n/a	[118]
β-glucan particles- aluminum salt	Hepatitis B	HBsAg	High anti-HBsAg IgG titer	n/a	[119]
β-glucan particles- PDMAEMA:PβAE/DNA polyplexes	Hepatitis B	HBsAg	Enhance transfection activity Great luciferase gene expression	n/a	[120]
**β-Glucan as Adjuvant**					
**Formulation**	**Application**	**Vaccine Antigen**	**Immune Response**	**Administration Route**	**Ref.**
n/a	High-risk neuroblasto-ma	GD2/GD3	High anti-GD2-IgG1 titer	Oral	[82]

HBsAg: Hepatitis B surface antigen; IgG: Immunoglobulin; IL: interleukin; MPs: Microparticles; MUC: Mucin; n/a: not applicable; NPs: Nanoparticles; OVA: Ovalbumin.

## Data Availability

Not applicable.
